# Supplementary material for: Global risk of wildfire across timber production systems
Source: Nat Commun. 2025 May 6;16:4204. doi: 10.1038/s41467-025-59272-6 (PMC12056198; doi:10.1038/s41467-025-59272-6)
Supplement: Supplementary file 1 — Supplementary Infomation [file 41467_2025_59272_MOESM1_ESM.pdf]

Global risk of wildfire across timber production systems

Supplementary Information

Christopher G. Bousfield[1,2*], Oscar Morton[1,2], David B. Lindenmayer[3], Adam F.A Pellegrini[1,2], Matthew G. Hethcoat[4] & David P. Edwards[1,2]

[1]Department of Plant Sciences and Centre for Global Wood Security, University of Cambridge, Cambridge, CB2 3EA, UK

[2]Conservation Research Institute, University of Cambridge, Cambridge, CB2 3EA, UK.

[3]Fenner School of Environment and Society, The Australian National University, Canberra, Australian Capital Territory, Australia

[4]Canadian Forest Service, Edmonton, AB Canada

[*]Corresponding author: cgb48@cam.ac.uk

List of Supplementary Items

Supplementary Figures 1-2

Supplementary Table 1

Supplementary Methods – Table 2

Supplementary Methods – Figure 3

Of the 17 countries that could be accurately matched, the three countries experiencing the highest total burned area in natural production forests were Brazil (3.7 M ha), the USA (3.1 M ha), and Australia (2.9 M ha) (Supplementary Figure 1). Collectively these three countries account for 61% of detected fire-induced losses of native production forest, globally. However, when considering what proportion of natural production forest burned in each country, Portugal suffered the highest losses, losing 7.8% in just 7 years, followed by Australia (5.3%) and Brazil (2.1%). In plantation forests, the three countries with the largest wildfire-induced losses were the USA (0.20 M ha), Australia (0.16 M ha), and Portugal (0.13 M ha), who lost 0.6%, 5.6%, and 15.2% of their national plantation forests, respectively (Supplementary Figure 1). Total burned area across these 17 countries was much higher in natural production forest largely due to its much larger extent, but on average, countries tended to lose a greater proportion of their plantation forest than natural production, with mean losses of 1.7% and 1.1%, respectively. However, these impacts are influenced by confounding variables that we account for through statistical matching.

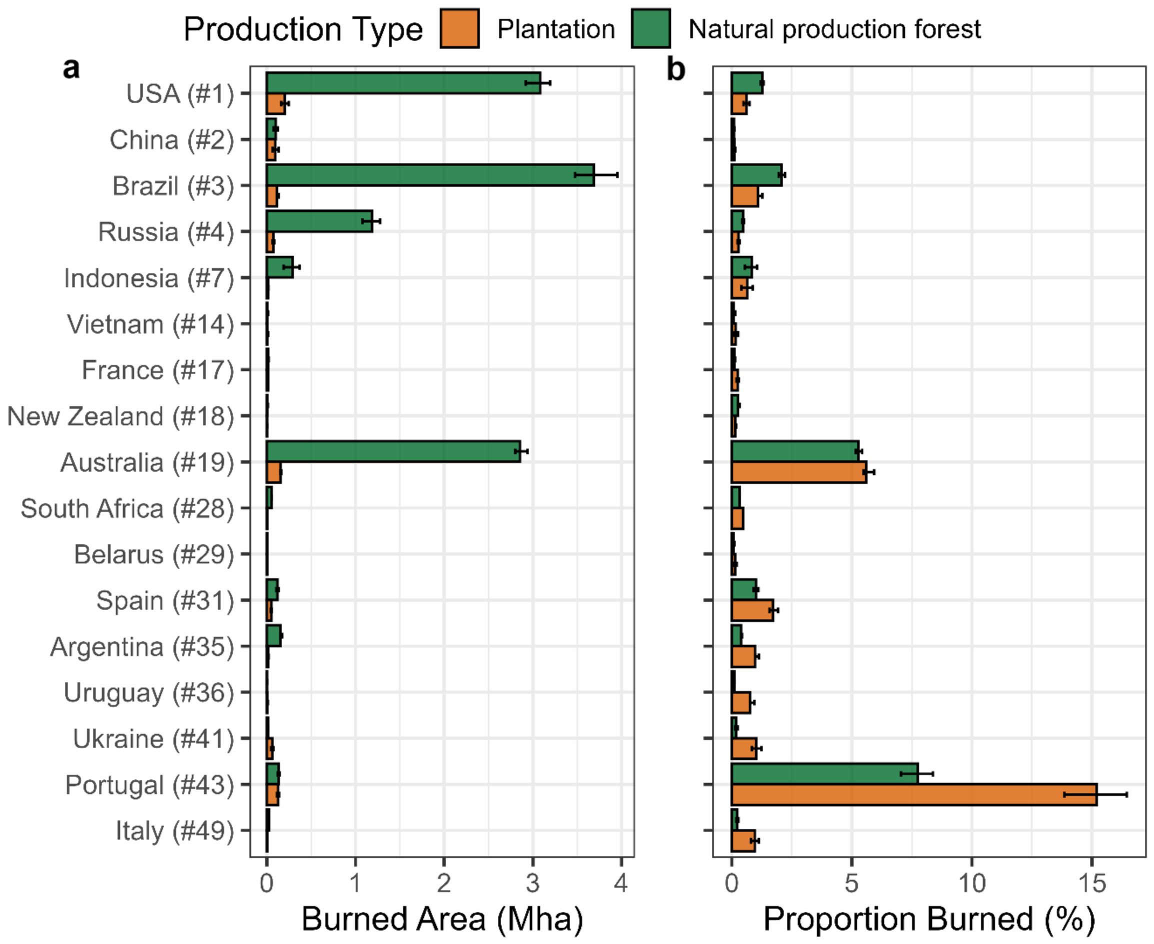

Supplementary Figure 1. National wildfire-induced losses of timber producing forest in matched countries between 2015 and 2022. Shown are the total burned area (a) and proportion burned (b) at the for the 18 countries where matching was possible. Losses in natural production forests are shown in green whilst losses in plantation forests are shown in brown. Error bars representing one s.e.m as per Tyukavina *et al.*

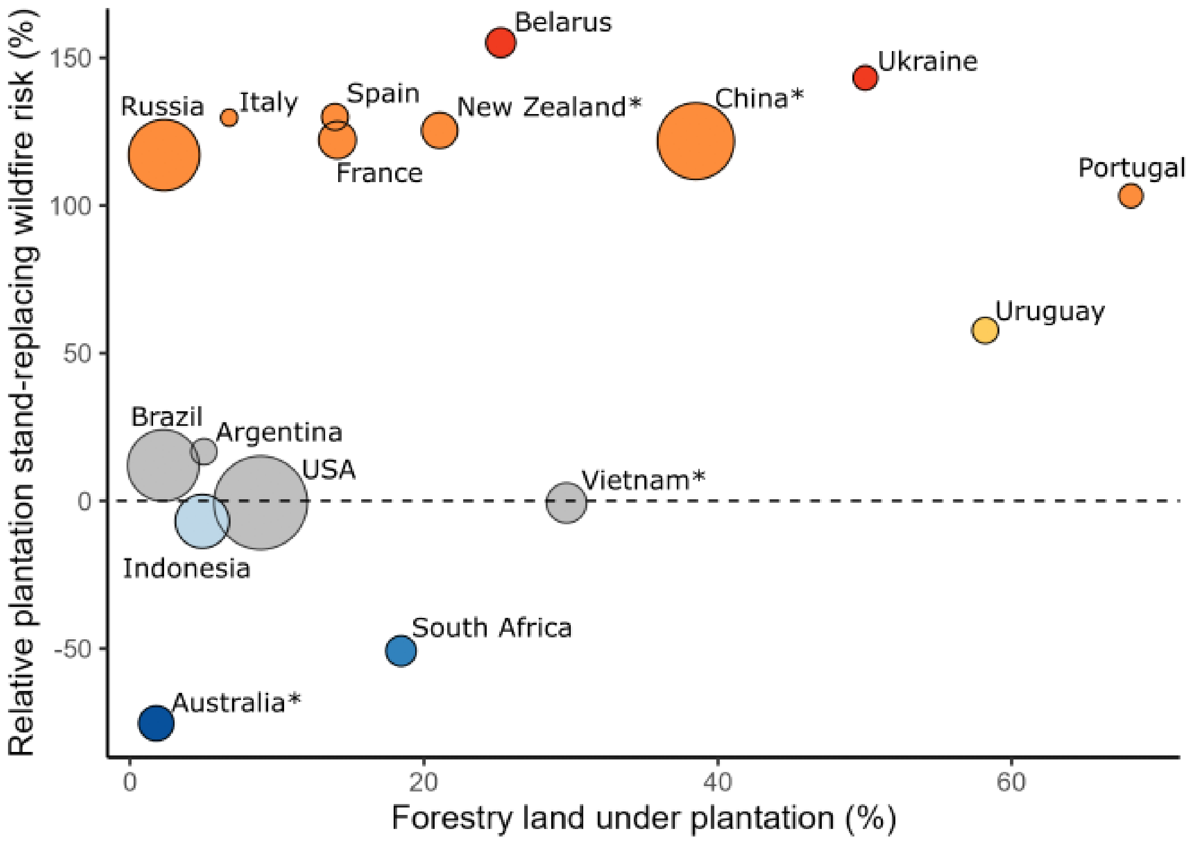

Supplementary Figure 2. National-level plantation wildfire risk effect size and percentage of timber-producing forest under plantation systems. Plantation stand-replacing wildfire risk represents the relative mean percentage change in wildfire probability in plantations compared to natural production forests under the same wildfire conditions. Points in orange to red represent increasingly high plantation wildfire effect (i.e. plantations are increasingly more likely to suffer stand-replacing wildfire than natural production forest), points in darker blue represent increasingly low plantation burn effect (i.e. plantations are increasingly less likely to suffer stand-replacing wildfire than natural production forest), and points in grey demonstrate countries with no significant plantation burn effect. Percentage of timber-producing forest under plantation systems is from the 2020 FAO Forest Resources Assessment[3]. Points are sized by their share of global timber production between 2015-2022 according to the FAO[21]. Countries with * are those that have banned native forest logging either at the national (China, Vietnam, New Zealand) or state level (Australia) and source the majority of their timber from plantations.

Supplementary Table 1. Comparison between total area of each timber production type from the Lesiv *et al.* dataset and the 2015 FAO Forest Resources Assessment. Percentages represent the percentage of a country's timber-producing forest that production type represents. 'Other naturally regenerated forest' best represents the natural production forest assessed in this study, but area extents are likely greater in the FAO Forest Resources Assessment due to this category also including recovering forests on agricultural land. *China is mapped by Lesiv *et al.* as having a combination of temperate and tropical plantations, so the value in this table is the combined extent of both plantation types.

Country	Natural production forest area (Mha)	Plantation area (Mha)	Plantation type	FAO FRA 2015 'Other naturally regenerated forest' area	FAO FRA 2015 'Planted forest'
**United States**	239,931 (88%)	33,097 (12%)	Temperate	208,431 (89%)	26,364 (11%)
**China**	106,499 (55%)	88,111* (45%)	Temperate	117,707 (60%)	78,982 (40%)
**Brazil**	177,251 (94%)	10,432 (6%)	Tropical	283,111 (97%)	7,736 (3%)
**Russia**	246,934 (90%)	26,528 (10%)	Temperate	522,372 (96%)	19,841 (4%)
**Indonesia**	34,418 (94%)	2,158 (6%)	Tropical	40,040 (89%)	4,946 (11%)
**Vietnam**	9,358 (63%)	5,451 (37%)	Tropical	11,027 (75%)	3,663 (25%)
**France**	14,990 (73%)	5,527 (27%)	Temperate	15,022 (88%)	1,967 (12%)
**New Zealand**	3,299 (69%)	1,494 (31%)	Temperate	5,905 (74%)	2,087 (26%)
**Australia**	53,809 (95%)	2,778 (5%)	Tropical	117,695 (98%)	2, 017 (2%)
**South Africa**	16,169 (94%)	992 (6%)	Tropical	6,531 (79%)	1,763 (21%)
**Belarus**	6,003 (60%)	3,945 (40%)	Temperate	6,323 (77%)	1,910 (23%)
**Spain**	11,814 (80%)	2,903 (20%)	Temperate	15,509 (84%)	2,909 (16%)
**Argentina**	40,143 (96%)	1,791 (4%)	Tropical	24,172 (95%)	1,202 (5%)
**Uruguay**	1,560 (58%)	1,150 (42%)	Tropical	470 (31%)	1,062 (69%)
**Ukraine**	6,383 (52%)	5,904 (48%)	Temperate	4,738 (49%)	4,860 (51%)
**Portugal**	1,759 (68%)	837 (32%)	Temperate	2,267 (72%)	891 (28%)
**Italy**	10,376 (96%)	449 (4%)	Temperate	8,565 (93%)	639 (7%)

Supplementary Methods

Supplementary Table 2. Data used, description and sources for the analysis.

**Variable**	**Description**	**Source**	**Use**
Forest management	identified as either "naturally regenerating forest with signs of management" (here natural production forest) or "planted forest"/"plantation forest" (here plantations) (100m)	Lesiv *et al.* 2022 https://www.nature.com/articles/s41597-022-01332-3	Treatment variable
Fire Occurrence	Occurrence of "stand-replacing wildfire" in a pixel in the years 2015-2022 (30m)	Tyukavina *et al.* 2022 https://www.frontiersin.org/journals/remote-sensing/articles/10.3389/frsen.2022.825190/full	Outcome variable
Elevation	SRTM 90m Digital Elevation Data	NASA STRM - https://srtm.csi.cgiar.org/	Covariate
Slope	SRTM 90m Digital Elevation Data	NASA STRM - https://srtm.csi.cgiar.org/	Covariate
Mean temperature of hottest quarter	Worldclim Bioclimatic variables (1km)	Worldclim https://www.worldclim.org/data/bioclim.html#google_vignette	Covariate
Mean precipitation of driest quarter	Worldclim Bioclimatic variables (1km)	Worldclim https://www.worldclim.org/data/bioclim.html#google_vignette	Covariate
Mean precipitation of wettest quarter	Worldclim Bioclimatic variables (1km)	Worldclim https://www.worldclim.org/data/bioclim.html#google_vignette	Covariate
Fire Weather Index (95th Percentile)	95th Percentile of ERA5-based Canadian FWI (28km)	Vitolo *et al.* 2019 https://www.nature.com/articles/s41597-020-0554-z	Covariate
Burned area history	Landscape level (1km) Mean annual burned area 2001-2014	Giglio *et al.* 2018 https://developers.google.com/earth-engine/datasets/catalog/MODIS_061_MCD64A1	Covariate
Landscape tree cover	Landscape level (1km) % tree cover in 2014	MOD44B Vegetation Continuous Fields V6 https://developers.google.com/earth-engine/datasets/catalog/MODIS_006_MOD44B	Covariate
Distance to road	Distance (m) to OSM major roads in 2016	Worldpop data https://hub.worldpop.org/geodata/listing?id=31	Covariate

Population density	Gridded population density for 2015 (1km)	GPW v.4 https://sedac.ciesin.columbia.edu/data/collection/gpw-v4	Covariate
Biome	WWF Terrestrial Ecosystems of the World	Olson *et al.* 2018 https://www.worldwildlife.org/publications/terrestrial-ecoregions-of-the-world	Covariate

Matching

To match native production points with plantation points that experienced similar wildfire-related conditions, we used the MatchIt package [47] to conduct statistical matching. Matching was done on a country by country basis, matching natural production forest points with plantation points (either "planted forest" or "plantation forest" as defined by Lesiv *et al.*[19] depending on the country) using nearest neighbour matching, and exact matching for biome. Matching was undertaken using four different matching methods (Propensity score matching with a 0.2 calliper, Propensity score matching with a 0.5 calliper, Mahalanobis distance matching with a 0.2 calliper, and Mahalanobis distance matching with a 0.5 calliper) using the following code:

## PSM – Calliper = 0.2 ##

myMatch <- matchit(treatment~ slope + elevation + pop.density + road.distance + hottest.quarter.temp + precip.wettest.quarter + precip.driest.quarter + tree.cover + burn.history + fwi.95, data=covariate.gridded.sample.native.vs.plantation, exact = 'biome', method='nearest', caliper=0.20)

## PSM – Calliper = 0.5 ##

myMatch <- matchit(treatment~ slope + elevation + pop.density + road.distance + hottest.quarter.temp + precip.wettest.quarter + precip.driest.quarter + tree.cover + burn.history + fwi.95, data=covariate.gridded.sample.native.vs.plantation, exact = 'biome', method='nearest', caliper=0.50)

## Mahalanobis – Calliper = 0.2 ##

myMatch <- matchit(treatment~ slope + elevation + pop.density + road.distance + hottest.quarter.temp + precip.wettest.quarter + precip.driest.quarter + tree.cover + burn.history + fwi.95, data=covariate.gridded.sample.native.vs.plantation, distance = 'glm', exact = 'biome', caliper=0.20, mahvars = ~ slope + elevation + pop.density + road.distance + hottest.quarter.temp + precip.wettest.quarter + precip.driest.quarter + tree.cover + burn.history + fwi.95)

## Mahalanobis – Calliper = 0.5 ##

myMatch <- matchit(treatment~ slope + elevation + pop.density + road.distance + hottest.quarter.temp + precip.wettest.quarter + precip.driest.quarter + tree.cover + burn.history + fwi.95, data=covariate.gridded.sample.native.vs.plantation, distance = 'glm', exact = 'biome', caliper=0.50, mahvars = ~ slope + elevation + pop.density + road.distance + hottest.quarter.temp + precip.wettest.quarter + precip.driest.quarter + tree.cover + burn.history + fwi.95)

Modelling

The matched datasets were then assessed for matching performance, with only countries with mean smd of <0.25 in all covariates taken through to the next step. The method returning the most countries

for further analysis was selected (PSM, calliper = 0.2 – 30 countries, Supplementary Figure 2). We removed countries where stand-replacing wildfires are extremely limited (< 0.1% burned area) and then pooled the matched datasets from each country into one dataset, and used the mgcv[58] package in R to fit a GAMM, fitting two separate models, one for matched natural production forests and "planted forests", and one for matched natural production forests and "plantation forests". To fit the GAMMs we used the following code:

# Natural production forests vs temperate "Planted forest" #

native.long.final.model.formula<- burn.num ~ treatment +s(x,y, k=250) + s(slope, bs='cr', k=20) + s(elevation, bs='cr', k=20) + s(pop.density,bs='cr', k=100) +s(road.distance, bs = 'cr', k=100) +s(tree.cover, bs='cr', k=20) +s(hottest.quarter.temp, bs = 'cr', k=100)+s(precip.wettest.quarter, bs = 'cr', k=100)+s(precip.driest.quarter, bs = 'cr', k=100) +s(fwi.95, bs = 'cr', k=100)+s(burn.history, bs = 'cr', k=10)+s(country, bs='re', by = country_dummy)+s(biome, bs='re', by = biome_dummy)+ s(treatment, biome, bs='re')+s(biome, tree.cover, bs='re')+ s(biome, country, bs='re', by = biome_country_dummy)

# Natural production forests vs tropical "Plantation forest" #

native.short.final.model.formula<- burn.num ~ treatment +s(x,y, k=250) + s(slope, bs='cr', k=20) + s(elevation, bs='cr', k=20) + s(pop.density,bs='cr', k=100) +s(road.distance, bs = 'cr', k=100) +s(tree.cover, bs='cr', k=20) +s(hottest.quarter.temp, bs = 'cr', k=100)+s(precip.wettest.quarter, bs = 'cr', k=100)+s(precip.driest.quarter, bs = 'cr', k=100) +s(fwi.95, bs = 'cr', k=100)+s(burn.history, bs = 'cr', k=10)+ s(country, bs='re', by = country_dummy)+s(biome, bs='re', by = biome_dummy)+ s(treatment, biome, bs='re')+s(biome, tree.cover, bs='re')+s(biome, country, bs='re', by = biome_country_dummy)

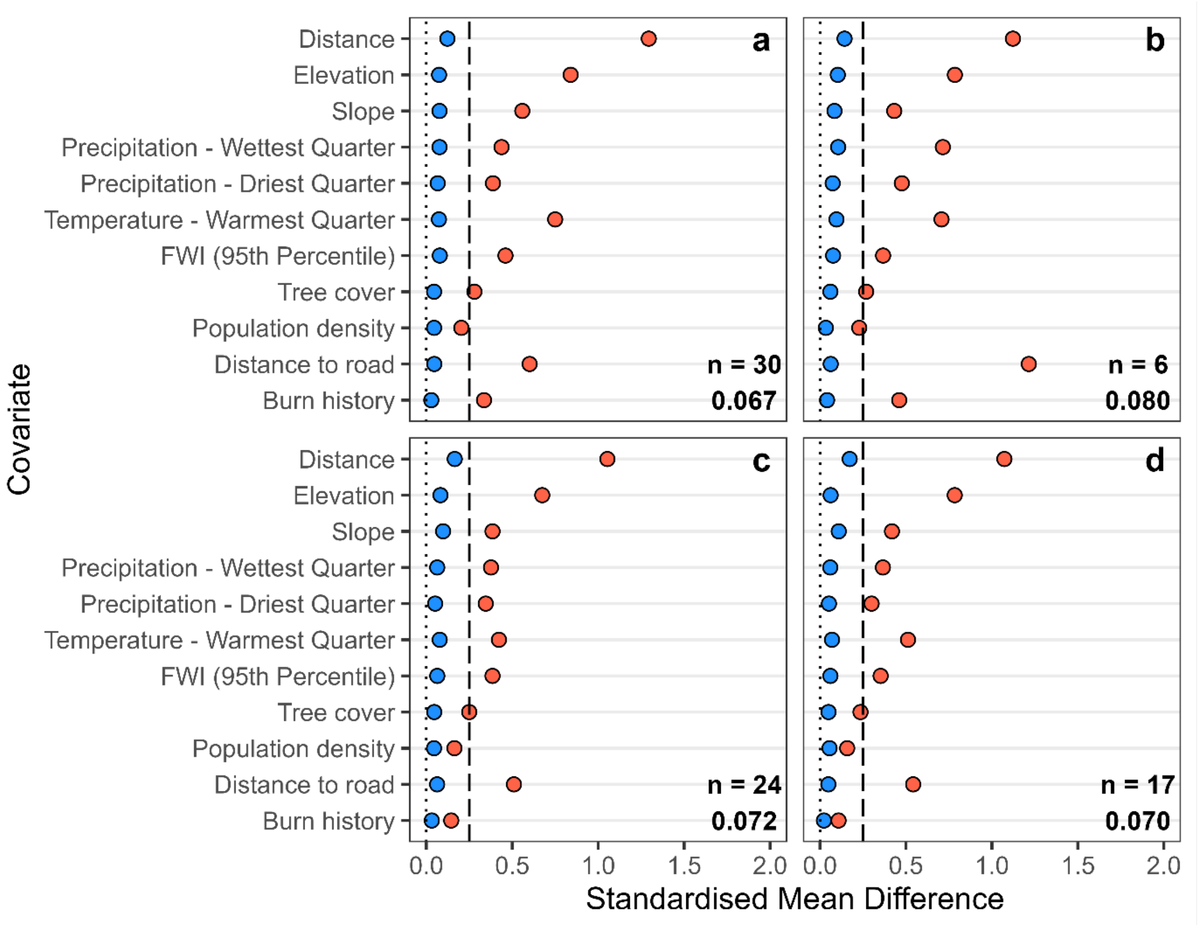

Supplementary Figure 3. Standardised mean differences from matching results for propensity score matching with a calliper of 0.2 (a) and 0.5 (b), and mahalanobis distance matching with a calliper of 0.2 (c) and 0.5 (d). Red dots represent the standardised mean difference (SMD) between treatment (plantation) and control (natural production) points before matching, and blue dots after matching. Dashed line represents a SMD of 0.25 (below this suggests good matching), dotted line represents a SMD of 0. Number of countries successfully matched with each method is displayed in bottom-right corner of each panel, alongside the mean SMD of all covariates.
